# Physicochemical and Sensory Stability Evaluation of Gummy Candies Fortified with Mountain Germander Extract and Prebiotics

**DOI:** 10.3390/polym16020259

**Published:** 2024-01-17

**Authors:** Aleksandra Vojvodić Cebin, Magdalena Bunić, Ana Mandura Jarić, Danijela Šeremet, Draženka Komes

**Affiliations:** Department of Food Engineering, Faculty of Food Technology and Biotechnology, University of Zagreb, Pierottijeva 6, 10 000 Zagreb, Croatia; avojvodic@pbf.unizg.hr (A.V.C.); mbunic@pbf.hr (M.B.); dseremet@pbf.hr (D.Š.)

**Keywords:** gummy candy, mountain germander, polyols, prebiotics, xylooligosaccharides

## Abstract

Health-conscious consumers seek convenient ways of incorporating different functional ingredients into their diets. Gummy candies are among the most popular confectionery products but generally regarded as nutritionally empty. A gelatin–sugar matrix, providing a highly appreciated sensory experience of sweetness and chewiness, could be used to deliver various bioactive compounds, especially those carrying an unpleasant taste. This work aimed to formulate gelatin gummies based on the mountain germander extract (MGe) as a source of phenylethanoid glycosides (PhEG). Sucrose and glucose syrup contents were partially or completely substituted with combinations of xylitol, maltitol and prebiotic poly- and oligosaccharides. Chemical, textural and sensory parameters were evaluated after production and 2 months of storage. Formulations containing fructooligosaccharides and xylooligosaccharides maintained a characteristic appearance during storage at all three levels of sugar (high, low and none), whereas inulin-added and plain (i.e., without prebiotic) candies suffered from mold contamination or appearance/textural changes. The color of the candies noticeably changed and appeared darker. The PhEG were shown to be stable during the candies’ production (approximately 90%) and generally maintained their contents during storage. Texture parameters, except hardness, exhibited high positive correlations and resembled the commercial product. Sensory-wise, a moderate bitterness intensity with a decreasing tendency, along with the high transparency and preservation of the characteristic shape facilitated high general acceptance. Gummy candies with prebiotics were shown to be a highly suitable matrix for the bitter MGe, delivering up to 40 mg of PhEG and 4.5 g of prebiotics in one serving size. This study provides a reference for implementing herbal extracts and emerging prebiotics (XOS) in functional confectionery.

## 1. Introduction

Modern consumers are increasingly oriented toward the health aspects of their nutrition. With the increasing demand for health-supporting supplementation, different ways and forms for the delivery of functional ingredients are being considered. Traditional oral administration forms, such as tablets or capsules, are being substituted for chewable dosage forms, with the advantage of flexibility in formulating more sensory-acceptable products that are easier to ingest [1]. Additionally, chewable products are more intuitively associated with food than tablets and capsules and provide a suitable nutritive context, especially for naturally derived functional ingredients. In this sense, gummy candies can play a significant role as food delivery forms for incorporating water-soluble functional ingredients. At the same time, their popularity among consumers could facilitate broader distribution and acceptance of such ingredients. Additionally, the expectancy of a sweet and pleasurable taste of a candy could have a significant role in masking or ameliorating possible undesired tastes of some functional ingredients [2].

Gummies are one of the most popular candy products, and especially fruitful when it comes to designing new products [3]. They are valued among consumers for their distinctive gummy texture and a variety of presentable shapes, aromas and colors. They are composed of sweeteners, hydrocolloids and additional ingredients, such as flavoring, coloring and acidulants [4]. The hydrocolloid used in their preparation is traditionally gelatin, providing clear gels and unique chewable texture, or it can be combined with other hydrocolloid-type ingredients, such as starch, guar gum, gum arabic, pectin and carrageenan, to produce an array of different yet similar textures to please the demanding market [5]. Gelatin is a water-soluble protein derived from acidic (type A) or alkaline (type B) pretreated collagen, usually from mammalian sources, such as porcine skin and bovine hide and bones [6]. It is commonly used as a food ingredient or additive for its gelling, thickening and stabilizing properties. The wide and versatile use of gelatin in the food industry is due to its availability, ease of use, thermoreversible gel properties and melt-in-mouth texture [7]. Although its use in food experiences limitations due to animal origin, religious constraints or possible allergenic potential [8], other nutritional concepts even favor the applicability of gelatin and collagen as potentially functional ingredients to promote the regeneration of soft tissues and bones, as well as hair and skin appearance and health [7,9,10]. 

Candy products, including gummy candies, are sometimes not fully recognized as a food category [11], probably due to their consumption pattern, which is closely associated with hedonic hunger and pleasure, combined with their poor nutritional value and high caloric load. Since they are consumed fairly often and by all age groups, improvements in their nutritional and functional aspects are important and can be pursued in many directions. In this sense, the confectionery industry has taken participation in the global turn toward the development of functional foods. Although the concept of functional candies is relatively new and still developing, the existence of sugar confections to target cough, sore throat, bad breath and nausea has been known for a long time [2]. The main directions nowadays include the development of low-calorie and low-sugar products and products enriched with phytochemicals, fruit-origin components, prebiotics and probiotics [8]. The incorporation of different herbal extracts into candy products presents a way of fortifying the product with phytochemicals, including polyphenols. The consumption of herbal extracts is highly associated with their expected medicinal properties. For that reason, the success of herbal confectionery is likely to be higher in markets where herbal remedies are well accepted and traditionally used [2]. Specific countries within the European market, such as Germany and France, for example, have shown to be inclined toward the use of herbal-based candy [2]. Mountain germander (*Teucrium montanum* L.) is a wild-growing herb from the *Lamiaceae* family, traditionally used in the Balkan region in folk medicine. Its medicinal potential is probably best reflected in the folk saying it “brings the dead back to life” [12]. Traditionally, mountain germander has been used for treating various health conditions, mostly digestive disorders, along with the reported diuretic and analgesic properties, as well as anti-inflammatory, antioxidative and antimicrobial effects [13,14]. In light of the recent COVID-19 pandemic, it is important to point out the traditional use of mountain germander in treating respiratory infections, such as productive cough (for its expectorant properties) or even in treating tuberculosis [13]. Mountain germander extracts are rich in phenolic compounds, including phenolic acids, flavonoids and phenylethanoid glycosides [12,13,15]. The latter are water-soluble compounds, and they have a chemical structure composed of three components, namely, phenylethyl alcohol, caffeic acid or a derivative, and a glycosyl group [16]. They compose an abundant group of molecules found across different plant genera and many of them having a remarkable bioactive potential. As reviewed in the work of Tian et al. [16], the bioactive properties of PhEG include antibacterial, antiviral, anti-inflammatory, analgesic, antitumor, antioxidant, neuroprotective, cardioprotective, anti-aging, hepatoprotective, nephroprotective, immunomodulatory, glucose regulatory other effects. Being the most abundant phenolics in mountain germander [12], its traditional use could be attributed to the benefits of PhEG. In fact, phenylethanoid glycosides have been identified as SARS-CoV-2 protease inhibitors [17,18], having a role as constituents of herbal preparations used to support drug treatment of COVID-19 in China [18]. This might provide insight into the role of mountain germander in treating pulmonary diseases. Although the incorporation of herbal extracts into candy products is mostly reserved for hard candies, it is interesting to investigate other candy types, such as gummies and jellies, as potential dosage forms. To the best of our knowledge, there are not any candy products containing mountain germander extract. 

Sugar content reduction in candy products is another way to achieve functionality. It is probably the most consumer-recognized functional feature due to the awareness of the role of excessive sugar intake in developing health problems. Indeed, many consumers are less likely to refrain from indulging foods if they are offered alternatives that are perceived as “better” yet taste the same as conventional products [19]. Sugar (i.e., sucrose) and glucose syrup are the main sweeteners in candy making, providing not only for sweetness but also for bulk and texture; hence, their removal and replacement is not easy [8]. Substitution with sugar alcohols (i.e., polyols) and fiber is the most common way of achieving this. Polyols carry fewer calories, contribute to establishing lower glycemic indices of food products and are noncariogenic, while most of them provide a body, mouthfeel and taste similar to that of sucrose [19,20]. Xylitol and maltitol are often used in sugar-free confectionery formulations. They exhibit sweetness close to that of sucrose [19]. In gummy candies, where maintaining texture is crucial, the incorporation of alternative sweeteners can notably alter gel strength, depending on the type and the amount of the sweetener [21]. Gunes et al. [5] provide a recent overview of the alternative sweeteners used in soft sugar confections, i.e., gummies and jellies. Within polyols, these included maltitol, maltitol syrup, isomalt, isomaltulose, xylitol and mannitol. Because of the large number of polyols as sugar-replacing options and the specificities of different candy types, new reference studies are much needed. The incorporation of soluble fiber with prebiotic properties into confectionery products, in addition to the reduction in sugar content, can provide the added value of gut health stimulation. Being the most studied and recognized as prebiotics, inulin and oligofructose (fructooligosaccharides (FOS)) are usually used in food products, confectionery included [22]. Other fiber sources in the context of gummy candies are being investigated as well, such as wheat fiber [23] and psyllium husk [24]. In recent years, lignocellulose-derived nondigestible oligosaccharides, such as xylooligosaccharides (XOS), have drawn much attention as emerging prebiotics [25,26]. They consist of a mixture of oligomers with a low degree of polymerization (usually 2–6) and are composed of linearly linked xylose units with β-(1-4)-glycosidic bonds. They can be obtained from xylan-rich plant residual materials, commercially from corn cobs [27], with alternative lignocellulosic sources increasingly being investigated [28] within the sustainable food production concept. XOS are characterized by high solubility in water, high thermal stability and resistance to hydrolysis in low pH media [29]. In food systems, they can exert different functions, including cryoprotective, texture modification and colloid-stabilizing roles [30]. Because of their moderate sweetness (30–60% sweetness of sucrose [29]), their use might be favored in food products that can be associated with a sweet taste. In addition to the mentioned technologically important aspects, the most significant are their prebiotic and bifidogenic properties for which XOS are predominantly used as functional prebiotic ingredients. Human intervention and animal studies have shown that prebiotics, in general, could modulate immune functions [31]. The combination of XOS and polyphenols in a formulated commercial product Slim (Plexus Worldwide) has shown immunomodulatory effects in the distant colon [32]. To the best of our knowledge, XOS usage in confectionery products is still in its infancy.

With the above stated, the aim of this work was to formulate gelatin-based gummies with varying sugar contents and fortified with mountain germander water extract, used as a liquid phase in the candies’ preparation. Sugar and glucose syrup were partially or completely replaced with polyols (xylitol and maltitol) and additionally with prebiotic fiber (inulin, FOS and XOS). The prepared candies were analyzed for chemical and sensory parameters after production and 2 months of storage to evaluate their stability and, moreover, the suitability of gelatin matrix as a carrier of functional components during preparation and storage. It was expected to formulate highly sensory-acceptable, innovative and functional candy products with the potential to support the immune system during respiratory infections due to the potential action of its functional ingredients—mountain germander extract and prebiotics. The sweet taste of the candy product, achieved by combining different sweeteners, could facilitate the acceptance of a highly bitter mountain germander extract. The incorporation of XOS would complement the existing knowledge on the usage of waste-materials-derived functional ingredients in food systems as a part of sustainable development in the food industry. Although many different functional ingredients have been evaluated for incorporation into candies, including vitamins, minerals and bioactives from various natural sources, the novelty of this study is in incorporating mountain germander extract and XOS in gummy candies, for which, to our knowledge, very little, if any previously reported data exist. In addition, it provides a reference for developing sugar-free candies using polyols and prebiotics, with the potential for commercialization. The incorporation of herbal extracts and prebiotics, as well as sugar reduction is highly compliant with the leading trends in developing functional confectionery.

## 2. Materials and Methods

### 2.1. Materials

Mountain germander (*Teucrium montanum* L.) was obtained from a local supplier “Ljekovito bilje Jerkin” j.d.o.o. (Zadar, Croatia). The material was collected in 2021 from the locality of Varivode, municipality of Kistanje (Dalmatia, Croatia). Areal plant parts were dried in the air, milled and sieved (≤450 μm) and used to obtain the water extract. A voucher specimen was deposited in the university herbarium of Herbarium Croaticum and registered within the Department of Biology, Faculty of Science, University of Zagreb (ID 75518). Sucrose (Agragold; Agana-Studen, Wien, Austria), glucose syrup—DE 36.0–40.5 (Fractal Colors; Csomar, Hungary), beef gelatin—bloom 220 (Nutrigold, Galleria Internazionale; Zagreb, Croatia), xylitol (Nutrigold, Galleria Internazionale; Zagreb, Croatia) and citric acid (Šafram; Zagreb, Croatia) were bought from a local store. Stevia powder (98% rebaudioside A and stevioside) was obtained from Wellgreen (Xi’an, China), maltitol from Iggos (Reire s.r.l.; Reggio Emilia, Italy), inulin and oligofructose from Orafti^®^GR (Beneo GmbH; Mannheim, Germany), xylooligosaccharides (XOS 95P, from corn cobs) from Shandong Longlive Biotechnology Co., Ltd. (Dezhou, China). The analytical standards of echinacoside (≥98%), verbascsoside (≥99%) and chlorogenic acid (≥95%) were purchased from Sigma-Aldrich (Merck Group, St. Louis, MO, USA), while glycerol, acetonitrile and formic acid from Fisher Scientific (Thermo Fisher Scientific Inc., Waltham, MA, USA). Ethanol (96%) was purchased from Kefo d.o.o. (Zagreb, Croatia). All chemicals used in the study were of p.a. (pro analysis) or HPLC quality (standards and solvents for mobile phases). 

### 2.2. Methods

#### 2.2.1. Preparation of Mountain Germander Extract

Ground dry mountain germander (*Teucrium montanum* L.) was mixed with boiling water at a 1:20 (*w*/*v*) ratio with occasional stirring for over 10 min. The extract was obtained by filtering (using commercial filter paper 235 for filtered coffee preparation). The filtrate was allowed to cool at room temperature and then stored at 4 °C for further use. Once prepared, the same extract was used to produce all candy formulations, i.e., preparation of the sugar syrup and gelatin solution.

#### 2.2.2. Production of Gummy Candy

The formulations of the gummy candies are provided in Table 1. The formulations varied in the sugar content: (i) “high-sugar” (HS) formulations prepared with sucrose and glucose syrup as sweeteners; (ii) “low-sugar” (LS) formulations that had half the sucrose replaced by xylitol; and (iii) “no-sugar” (NS) formulations in which sucrose was replaced by maltitol and glucose syrup by xylitol. These formulations carried the denotation “b”, for “basic”, in subscript. In addition, inulin, fructooligosaccharides and xylooligosaccharides were added to other gummy formulations (as denoted by the subscript), replacing one-third of the sucrose in the HS, two-thirds of the sucrose in the LS and one-third of the xylitol in the NS formulations.

All gummy candy formulations were prepared in the same manner. The three major components—gelatin solution, sugar syrup and citric acid/stevia solution—were prepared separately and then combined to produce a gummy candy mass that was conditioned and molded. The production stages were as follows. 

##### Preparation of Gelatin Solution

Ground gelatin (30 g) was thoroughly mixed with the extract (60 mL) (Table 1) in a beaker and left still at room temperature for 10 min to allow the gelatin particles to hydrate (the ground gelatin quickly swelled and could not be further mixed). Afterwards, the swollen gelatin was placed in a water bath (Inko, VK2; Zagreb, Croatia) heated at 60 °C to dissolve for approximately 20 min while occasionally stirring with a spatula. The gelatin solution preparation was coordinated with the cooking of the sugar syrup and was kept warm (at 60 °C) until combined with the syrup. Fresh gelatin solution was prepared for each batch of candies.

##### Preparation of Citric Acid and Stevia Solution

Citric acid (3 g) (and stevia (0.3 g) where needed) was mixed with approximately 1.5 mL of the extract in a small beaker and completely dissolved by stirring and gentle heating (the beaker was occasionally placed in a water bath at 60 °C for a short amount of time to help it dissolve). The solution was left still at room temperature until used. The citric acid/stevia solution preparation was coordinated with the preparation of the sugar syrup, so the solution was ready to mix with the syrup when the cooked syrup cooled down. A fresh solution of citric acid/stevia was prepared for each batch of candies.

##### Preparation of Sugar Syrup 

Mountain germander extract (60 g) was heated on a cooking plate in a 0.4 L metal pot. When bubbles started to form on the bottom, the carbohydrate ingredients for each formulation (Table 1) were slowly added, one by one, while stirring with a silicon spatula and ensuring not to splash. The stirring continued until the carbohydrate ingredients were completely dissolved and the solution started to boil. Then, the heating was reduced to low–intermediate, a temperature probe (Testo, 108-2; Titisee, Germany) was inserted and the solution was left still and uncovered to steadily simmer. The monitored boiling temperature slowly and steadily increased. The process continued until reaching a thick syrup consistency, which took approximately 15 min. During this time, the sugar concentration was occasionally checked using a digital refractometer (Kern optics, ORF 85BM, Kern&Sohn GmbH; Balingen, Germany) to ensure it reached 85 °Bx, at which point the monitored temperature (Testo, 108-2; Titisee, Germany) was 115–120 °C, depending on the ingredients (formulation).

The obtained sugar syrup was removed from the heat and left to cool on the counter, at room temperature. The cooling proceeded until reaching approximately 75 °C. 

##### Preparation of Gummy Candy Mass and Formation of Candies

Once it reached approximately 75 °C, the syrup was cool enough to add citric acid/stevia and gelatin solutions, one right after the other (both simultaneously prepared while the syrup was cooking). The combined solution was carefully stirred to ensure proper homogenization but also to avoid excessive aeration. 

The obtained gummy candy mass was transferred in a beaker and tempered at 60 °C in a water bath for 15 min to clarify the mass from air bubbles (collected on the top and scooped aside before pouring into the molds). The warm mass was carefully poured into bear-shaped silicon molds using a plastic Pasteur pipette with a large opening and left to cool for 1 h at 4 °C and then for the next 20 h at room temperature while covered with parchment paper. The formed gummy candies (i.e., gummy bears) were taken out of the mold using lightly oiled (cooking oil) gloves.

#### 2.2.3. Stability Study

The prepared candies were divided into 2 equal portions (for each formulation) and packed into transparent polypropylene zip-lock bags (commercial bags used to store food). The bags were stored at room temperature and exposed to combined natural and artificial light. The storage conditions most likely reflected those in retail (standing on a shelf). All candies were stored for 2 months (60 days). 

#### 2.2.4. Determination of Dry Matter

Dry matter was determined gravimetrically upon drying at 105 °C until a constant mass [33]. An approximately 4 g (equivalent to 2 gummy bears) sample size was used. The analysis was performed in triplicate, and the results are expressed as the means with the standard deviation.

#### 2.2.5. Determination of Water Activity

The water activity was determined using a HygroPalm GP23 (Rotronic, Switzerland) instrument. Approximately 10 g of gummy bears were cut into small pieces using scissors and left to equilibrate at room temperature for at least 30 min in closed measuring vials. After equilibration, the samples were quickly transferred for measurement using the a_w_Q mode. The measurements were carried out in triplicate, and the results are expressed as the mean values with the standard deviation. 

#### 2.2.6. Determination of pH Value

The pH value was determined in a candy solution using a pH-meter (Five Easy FE20, Mettler Toledo; Zürich, Switzerland), following a procedure of Delgado et al. [34], with modifications. For that purpose, approximately 4 g of candies (equivalent to 2 gummy bears) were mixed with 2 mL of demineralized water and heated at 50 °C for 30 min (until dissolution). The dissolved candies were quantitatively transferred to a 10 mL flask and left at room temperature for a short time (15 min) to cool. The measurements were performed in triplicate and the results are expressed as the means with corresponding standard deviation. 

#### 2.2.7. HPLC Analyses

##### Sample Preparation

Prior to the analyses, gummy bear samples were specifically prepared to remove the gelatin. To the glass tubes containing approximately 2 g of gummy bears (equivalent to 1 candy), 500 μL of chlorogenic acid solution (500 μg/mL) and 500 μL of glycerol solution (500 mg/mL), serving as internal standards for polyphenols and sugars, respectively, were added. Capped tubes were heated at 50 °C for 30 min (Dlab BH120-S; Beijing, China) and occasionally mixed by vortex (Dlab MX-S; Beijing, China). A 4 mL portion of 96% ethanol was added to the dissolved candies while vigorously mixing, followed by another 4 mL. The tubes were kept at 4 °C for 30 min to induce gelatin precipitation. The precipitate was removed by centrifugation (4 °C, 10 min, 4000 rpm) (SL 8R centrifuge with a fixed-angle rotor; Thermo Scientific, Whaltam, MA, USA) and rinsed with 5 mL of 78% ethanol. The supernatants were collected in a round-bottom flask and evaporated using a rotary vacuum evaporator (RV-8, IKA; Staufen, Germany) at 40 °C and 100 mbar until the ethanol was removed. The evaporated extract was quantitatively transferred to 5 mL flask with demineralized water. Sample preparation was performed in triplicate. 

##### Analysis of Polyphenols by Reversed-Phase HPLC 

The analysis was performed using an Agilent 1200 Series chromatograph (Agilent Technologies, Santa Clara, CA, USA) coupled with a Zorbax Extend C18 (4.6 × 250 mm, 5 μm) column (Agilent Technologies, Santa Clara, CA, USA) and DAD detector. The mobile phase comprised a binary system: A—0.1% (*v*/*v*) formic acid in water and B—0.1% (*v*/*v*) formic acid in acetonitrile. Gradient elution at a 1 mL/min flow rate was performed as follows: 0 min—7% B, 5 min—7% B, 45 min—40% B, 47 min—70% B, 52 min—70% B + 10 min of equilibration to start the mobile phase composition. The column was thermostated at 25 °C, and the injection volume was 5 μL. The chromatograms were recorded at 320 nm and the absorption spectra in the 190–600 nm range. The peaks were identified by a retention time comparison with known standards. Internal standard calibration curves using chlorogenic acid (50 μg/mL) were established for verbascoside and echinacoside in the 10–100 μg/mL concentration range. The rest of the phenylethanoid glycosides (identified by a comparison of the UV-VIS spectra) were reported as echinacoside equivalents because of the lack of corresponding standards. The results are expressed as the means with the standard deviation.

##### Analysis of Sugars by Ligand-Exchange HPLC

The analysis was performed on Agilent 1200 Series chromatograph (Agilent Technologies, Santa Clara, CA, USA) coupled with Zorbax Hi-PlexCa (7.7 × 300 mm) column (Agilent Technologies, Santa Clara, CA, USA) and RI detector. The elution was isocratic (30 min) at 0.6 mL/min, using demineralized water as the mobile phase. Column temperature was set at 80 °C, injection volume was 10 μL. RI detector was set at 40 °C and the signal recorded at 2 s response time. The peaks were identified by retention time comparison with known standards. Internal standard calibration curves using glycerol (50 μg/mL) were established for sucrose, glucose, fructose, xylitol and maltitol. The results are expressed as the means with the standard deviation.

#### 2.2.8. Texture Analysis

Texture profile analysis (TPA) was performed using TA.HD.plus Texture Analyser (Stable Micro Systems; Godalming, UK) equipped with a load cell of 5 kg. All measurements were performed at room temperature. The test was conducted with a 50 mm cylindrical plate. The conditions comprised 2 consecutive cycles of compression to a 1 mm distance upon a trigger force of 509.9 g, with 5 s between cycles. The test speed was 5 mm/s. From the force–time curve, the following parameters were quantified: hardness, resilience, cohesiveness, springiness and chewiness. Data were obtained using Texture Exponent (v6.2) (Stable Micro Systems; Godalming, UK) with TPA macro setup. All measurements were performed in triplicate, and the results are expressed as the means with the standard deviation. 

#### 2.2.9. Color Measurement

The color parameters were measured using a benchtop colorimeter Chroma meter CR-5 (Konica Minolta; Chiyoda City, Tokyo, Japan). The top measurement port was adjusted to 8 mm. The evaluated color parameters were L* (lightness), a* (±red–green) and b* (±yellow–blue). Preliminary measurements using dark chamber cover and daylight illumination did not show a difference in the obtained color parameters; hence, all measurements were performed in natural daylight. The measurements were performed in triplicate, and the results are expressed as the means with the standard deviation.

#### 2.2.10. Sensory Evaluation 

The sensory analysis was performed by a trained internal panel consisting of 10 females (23–64 years old), of which 8 were employees of the Faculty of Food Technology and Biotechnology, with previous experience in the sensory evaluation of different food products, and 2 were students studying food technology within the same faculty. A descriptive analysis was performed as an intensity evaluation of the defined parameters: transparency (as an appearance descriptor), sweetness and bitterness (as taste descriptors) and hardness (as a texture descriptor) on a scale of 1–9, where 1 denoted “no intensity”, 5 “moderate intensity” and 9 “extreme intensity”. In addition, the panelists evaluated “General acceptance” on a hedonic scale of 1–9, where 1 denoted “not acceptable”, 5 “moderately acceptable” and 9 “extremely acceptable”. The samples were divided into groups of 3, with 1 group of the basic formulations and 3 groups of the prebiotic formulations according to the sugar content. All samples were coded and evaluated under daylight and at room temperature. The panelists were deployed into separate cubicles and were offered plain rice crackers and still water in between samples to neutralize the taste. 

#### 2.2.11. Statistical Analysis

The statistical analysis was performed using a paired *t*-test at a level of α = 0.05 (SPSS 17.0; Microsoft, Redmond, WA, USA) to evaluate the difference between the means for each candy formulation over the storage time. In the texture analysis, the Pearson correlation coefficients between the parameters evaluated in the TPA analysis and sensory hardness were determined for the samples after their production and 2 months of storage, separately. 

## 3. Results and Discussion

In this study, gelatin gummy candies were formulated with various sugar contents and fortified with mountain germander water extract and prebiotics. The extract is rich in phenylethanoid glycosides, which are known to possess many bioactive properties. The incorporation of prebiotics, including emerging prebiotics—xylooligosaccharides—additionally supported the potential functionality of the formulated candy toward the sustainability of gut health and further to an overall possible effect on the immune system. Formulations with various sugar contents will enable the delivery of these beneficial components to a larger group of consumers, as well as possibly reduce or exclude the negative perception of the candy product, in general, resulting from the supposed sugar intake. 

### 3.1. Visual Appearance of the Candy

**Figure 1:**
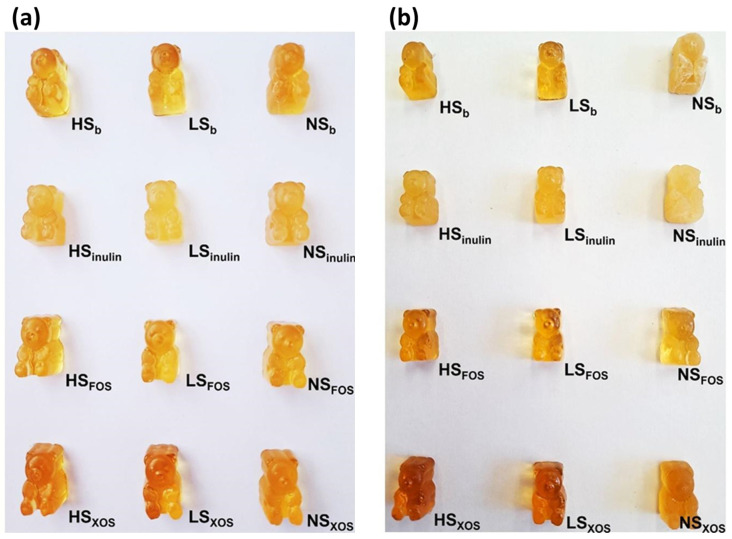
The appearance of the gummy candies after production (**a**) and 2 months of storage (**b**) (HS—“high-sugar” formulation; LS—“low-sugar” formulation; NS—“no-sugar” formulation; b—basic formulation; FOS—fructooligosaccharides; XOS—xylooligosaccharides).

The candy formulations after production and after 2 months of storage are presented in Figure 1. After production, all candies were easily removed from the silicon molding and retained the characteristic shape. The formulations containing inulin appeared the least transparent, and the NS formulations as less transparent in comparison to their sugar-containing counterparts. The color of the candies was in shades of yellow/amber, resulting from the mountain germander extract. The perception of the color might have been influenced by the sugar composition, as the formulations differed in transparency. The formulations containing XOS appeared a bit darker than the others. After 2 months of storage, the majority of the candies retained their shape and appearance. The NS_b_ and NS_inulin_ formulations exhibited shape and texture changes over time, which were observed visually (Figure 1). In NS_b_, the surface shrank and appeared mattified and white on the edges, which was probably due to the crystallization of xylitol. When only xylitol was used as a sweetener in the NS_b_ formulation (preliminary), the crystallization started to occur after approximately 5 days and was more pronounced. Substituting a part (two-thirds) of the xylitol with maltitol resulted in a more stabilized formulation, but it did not prevent eventual crystallization over time. It could not be distinguished whether the crystallization was due to xylitol or maltitol. However, it was interesting to observe that this effect did not occur in the XOS- and FOS-containing NS candies in which the maltitol content was further reduced. On the other hand, in the NS_inulin_ formulation the candies lost the characteristic shape on the surface as the surface became softer, possibly due to the start of the loss of gel strength. The XOS-containing formulations seemed to darken over the 2 months of storage. 

### 3.2. Dry Matter Content, Water Activity and pH Value

The dry matter of the candy formulations ranged approximately from 70 to 76%. The dry matter content during preparation was monitored using a digital refractometer and by reproducing the cooking time, as well as heat intensity. Since the HS and LS formulations contained glucose syrup and, consequently, more moisture in the initial formulation, the NS formulations resulted in higher dry matter content (approximately 75–76%) for the same preparation protocol applied. The typical moisture content of gummy candies is between 10 and 20% [5]. The herein obtained higher moisture content (Table 2) was directly related to the cooking duration and the amount of extract used in the gelatin solution preparation. Since the refractometer was calibrated up to 85 °Bx, this was the limit to monitor while cooking the sugar syrup, while the subsequent addition of gelatin solution increased the moisture content of the final candy mass. The moisture content of candies is a very important quality parameter, as it majorly affects texture and the overall stability of the product, including microbial contamination [35]. The NS formulations containing inulin and XOS exhibited a significant increase in dry matter content over time, implying a higher risk of the NS formulations drying out. Indeed, the glucose syrup present in the HS and LS formulations might have contributed to the better retention of moisture in those formulations, as it can acts as a humectant [36]. In addition to the dry matter content, the water activity (a_w_) value was also determined as an indicator of the stability of the product (Table 2). The a_w_ value of confectioneries, including gummies, is affected by their moisture content, as well as sugar profile. In addition, the contents of salt and components acting as humectants also affect the a_w_ value [35,37], which is defined as the ratio of the vapor pressures of the product to plain water, or the ability of the molecule to escape the surface of the product [35]. The a_w_ values of the soft confectionaries, including gummy candies, fall between 0.45 and 0.75 [5,36]. The herein obtained values of 0.69–0.79 were in the upper limit of the expected range, which probably resulted from the somewhat higher moisture content, in addition to the compositional profile of each formulation. According to Plotnikova et al. [37], the formulated gummy candies fall into the category of products with an intermediate humidity, for which oxidative, microbiological and enzymatic processes are possible. For the obtained values, the microbiological stability implied the susceptance to the growth of molds and yeasts [35]. Indeed, in some formulations, namely, HS_b_, HS_inulin_ and LS_inulin_, mold growth was observed on the inside corners of the packaging after 2 months of storage at room temperature, and for safety reasons, these formulations were excluded from sensory evaluation. Opposite to the dry matter content, the a_w_ values were shown to change significantly during storage, although this change was rather small and in most cases the a_w_ value increased, indicating a somewhat increased water mobility within the candy. Although the a_w_ values were higher than expected for this type of candy, sufficient stability might have been achieved by the addition of citric acid, which lowered the pH of the prepared gummy candies. Normally, citric acid (or other types of food-appropriate organic acids) is added to balance the sweetness and other flavors, but it can express other benefits as well. The pH value of the candies ranged between 3.54 and 3.91 and was maintained during storage. Products with a pH from 5 to 7 carry a higher risk of contamination with pathogenic microorganisms [36]. The herein obtained values are in accordance with the typical pH of gummies of 3 to 5 [38] (Table 2).

### 3.3. Content of Phenylethanoid Glycosides

The contents of phenylethanoid glycosides (PhEG) were established through the determination of echinacoside and verbascoside as available standards, while other phenylethanoid glycosides were identified in previous work by Mandura Jarić et al. [12], including teupolioside, stachyoside A and poliumoside, using advanced analytical methods, as described in the mentioned work. The latter were expressed as echinacoside equivalents due to its dominant contribution to the total phenylethanoid content. All of the phenylethanoids present in the gummy bears directly derived from the mountain germander water extract. Although the extract contained other phenolics due to the bioactive potential of PhEG, with echinacoside [39], in particular, being the most or among the most abundant of the PhEG in *T. mountanum*, these compounds were considered to be the most valuable bioactive constituents of the extract. The contents of echinacoside, verbascoside and the sum of other mentioned phenylethanoid glycosides are presented in Table 3. 

The obtained values showed a relatively equal distribution of PhEG in the prepared formulations. The original extract used in the candies’ production contained 1603 μg/mL of echinacoside, 200 μg/mL of verbascoside and 1292 μg/mL of other PhEG as echinacoside equivalents. Taking into account the volume of the extract used, as well as the calculated total mass of the candies, the obtained results show the high stability of the PhEG during production (minimally 90%), during which high-temperature exposure, along with the risk of hydrolysis upon the addition of citric acid, probably carried the greatest risk for their degradation. It was reasonable to expect that the contents of PhEG were preserved during their extraction from the starting material (i.e., dried mountain germander), since lower temperature and shorter exposure time was employed in comparison to the candies’ production. PhEG have shown to be fairly stable in temperatures used for foods, namely, confectionery processing, thus enabling their bioactive potential in the final product. The contents of PhEG were maintained throughout the storage period (Table 3), with only separate cases, such as HS_b_, LS_b_, and NS_inulin_, exhibiting significant reduction over time, although still rather low. This could be due to the start of microbial contamination, as observed. This result of highly preserved contents of PhGE was irrespective of the sugar content and the presence or type of prebiotic in the formulation. The presented data identify PhEG as being stable under exposure to heat (15 min exposure above 100 °C) and oxidation, as evidenced in the gummy candies, which is important for their potential application in other food systems. The consumption of one 30 g serving size of the candies [40] contributes to 15.1–19.9 mg of echinacoside, 2.2–2.8 mg of verbascoside and 14.3–17.2 mg of other PhEG. Phenylethanoid glycosides are being investigated for their remarkable pharmacological potential, since they are a part of different herbal preparations traditionally used in folk medicine. Among the many reported beneficial health properties, PhEG also exhibit immunomodulatory potential [16]. Although the biological activity of plant extracts, in general, is the result of many different constituents, with many of them working together, PhEG have shown potential in treating pulmonary infections [18]. Therefore, the precise identification of PhEG, together with the evaluation of their bioactive potential, could certainly facilitate the preparation of target formulations containing herbal extracts to support, for instance, the immune system. Further, being secondary metabolites, the contents of PhEG can vary with respect to the plant origin and growing conditions. Therefore, the precise identification and standardization of PhEG contents are needed for producing candies of unified quality. 

### 3.4. Color

The color of confectionery products is a strong driver of their overall acceptability, since it impacts on taste perception and threshold and the expected pleasant sensation upon consumption [5]. The color of the prepared gummy bears in this paper was measured using a colorimeter and was defined within the CIEL*a*b* color space (Table 4). The color derived exclusively from the mountain germander extract. The candies were in shades of yellow/amber (Figure 1), resulting in a lightness (L*) range of 26.69–37.34, redness range of 1.72–5.31 and yellowness range of 9.00–17.69, hence, leaning toward red and yellow shades. The observed color seemed adequate and representative of the extract contained in the formulation, so there was no need for adding colorants. The total appearance of the candies was also affected by their sugar composition, as some formulations were more transparent and glossier than others, so the perception of their color also varied. After 2 months of storage, the color of the candies changed. Visually, the candies appeared darker (Figure 1). Indeed, lightness significantly changed in half of the formulations and only in the NS_b_ sample it increased, while in the other samples it decreased. The increased lightness in the NS_b_ sample is probably due to the observed crystallization (Figure 1). In some samples, along with the lightness change, there were also significant changes in one of the chromatic coordinates (a* or b*), thus additionally contributing to the overall change in the color (ΔE) of the candy samples. The ΔE parameter was calculated for each formulation over the storage time to quantify the change in color in relation to the changes in L*, a* and b* [41]. The ΔE ranged between 1.83 (NS_inulin_) and 11.28 (NS_b_), but generally, it was observed to range between 3 and 6. It is considered that a color difference (ΔE) of at least 2.2 is needed to make two images distinguishable [42]. Regarding the supposed thresholds for perceiving the color difference, as reported in a paper by Mokrzycki and Tatol [41], there was a clear difference in the color (3.5 ≤ ΔE ≤ 5) that was noticeable by the observer (ΔE ≥ 5) for the majority of the candy formulations as a function of time. The least color difference was observed in the inulin-containing formulations (up to 3.45), which was probably influenced by their initially opaque appearance. The highest color difference was observed for the NS_b._ sample, most probably reflected from the observed crystallization, as mentioned earlier. Although the color of the candies changed, it still seemed natural and suitable for the product, and the observed difference can be regarded as tolerable for this purpose. The mechanism of the observed color change is too difficult to predict or explain within the scope of this paper, taking into account the complex nature of the employed extract, the possible interactions of its constituents with sugars and gelatin, and the storage conditions.

### 3.5. Sugar Profile and Content

An HPLC analysis of the sugars was performed to evaluate the stability of the sugar ingredients under the conditions of high-temperature exposure and presence of citric acid in the candies’ production. The analysis revealed the presence of added sugars: sucrose, xylitol and maltitol, whereas glucose and fructose were not added, per se, but resulted from their native presence in the glucose syrup or FOS, respectively, or as hydrolysis products of sugar ingredients, to some extent. Sucrose was also found in inulin (Table 5), so it contributed slightly to the total sucrose content in the formulations containing inulin (approximately 3–9% of the obtained result). Sucrose was also observed in the NS_inulin_ formulation, accounting for approximately 1%. Although no sucrose was used in the NS formulations, the final sucrose content could imply that this formulation no longer qualifies for the “sugar-free” category, which comprises products containing less or equal to 0.5% of sugar content [43]. This could be a limiting factor for the content of inulin to be used in sugar-free candy formulations, of course, depending on the quality of the inulin as a raw material. Hence, determining the sugar content of the ingredients is an important step in defining candy formulations within a specified category with respect to sugar content. This also applies to the use of FOS in candy formulations, since they contained 4.2% of fructose (Table 5). Another aspect to account for in discussing the sucrose content of the prepared candies is the coelution of sucrose and maltose. Because of the chromatographic limitations of the analysis, sucrose and maltose could not be efficiently separated. Maltose in the prepared gummies arose from its presence in the glucose syrup, where it accounted for approximately 11.5% (Table 5). The maltose content was estimated to be, on average, 2.1% in the gummy candy formulations containing glucose syrup. Alongside maltose, glucose was also markedly represented in the glucose syrup, up to 15.4% (Table 5). The total content of glucose was a combination of the glucose content from the glucose syrup and the glucose liberated (alongside fructose) from the inversion of sucrose or other ingredients (e.g., sugar syrup). Sucrose inversion during production and storage can have a significant impact on the quality of the candy. In order to minimize sucrose inversion, citric acid was added in the late stages of the candies’ production. From the obtained results (Table 5), it was calculated that no excessive inversion of sucrose occurred during the production of the gummy candies. The fructose content was used as an indicator of sucrose inversion, with the exception of the FOS-containing candy, in which fructose was calculated to contribute approximately 0.6% of the candy. Sucrose inversion was observed in the HS formulations and exhibited up to 3.3% of the sucrose content (HS_b_). The observed changes in the fructose content over time could imply that additional inversion occurred during storage. 

In fructan-containing formulations, the fructose content was a combination of the fructose from the FOS, fructose from the sucrose inversion, and probably from fructan hydrolysis to some extent. It is important to observe that the XOS did not include xylose (Table 5), and that they remained stable during the candies’ production and storage, i.e., no hydrolytic effects were observed. Therefore, it is reasonable to expect that the complete prebiotic potential of the used XOS was preserved. The same was observed for the FOS and inulin, although because of the presence of sucrose and fructose in their composition, the expected contents of prebiotic oligosaccharides was lower than the amount of added inulin or FOS. In comparison to the latter, XOS exhibited a lower effective dosage. As reported, the prebiotic effects of XOS can be observed at a dosage of approximately 3 g/day [44]. According to this and taking into account that the added amount of prebiotic ingredients was 14–15% per candy mass, the consumption of one serving size would satisfy the effective dosage intake for XOS. Additionally, all prebiotic-containing formulations could carry the claim of “high in fiber” due to their content being higher than 6 g per 100 g of the product [44]. The consumption of one serving size would contribute to approximately 20% of the recommended daily intake of fiber for adults [45].

Regarding the polyol content in the candy formulations, the herein used contents of xylitol (approximately 21–23% per candy mass) and maltitol (either approximately 30% or 46% per candy mass) are comparable to those found in the formulations of commercially available polyol-containing gummy candies [46,47]. As previously reported, maltitol syrup is a suitable polyol ingredient in sugar-free gummy candies, since it enables the full gelling power of gelatin. However, there is a risk of crystallization if the syrup is high in maltitol [19]. This could be the cause of the observed crystallization in the NS_b_ formulation during storage, whereas the reduction of its content with the substitution of prebiotics resulted in a more stable formulation (Figure 1). Xylitol crystallization or cocrystallization could not be excluded, especially because of the trial batches (not reported) in which xylitol was used as the only sweetener and crystallization occurred after 5 days. The relatively high final moisture content of the produced candy might have triggered the crystallization in all these cases.

The determined contents of all evaluated sugars corresponded to the formulations (Table 1). Comparing the results of the sugar analysis after 2 months of storage, the contents of all measured sugars were generally maintained. Although the differences in some cases were significant, they were fairly low and could be ascribed to the robustness of the method, which is yet to be validated, rather than a true change in the contents.

### 3.6. Texture Analysis

Generally, the texture of confectionery products, particularly, soft candies, is dependent on multiple factors, including the nature of the ingredients, the formulation and the production process itself. In gummy candies, the most influential factors are the amount of water in the product and the type and amount of hydrocolloid used for gelation [5]. Normally, lower water contents and higher hydrocolloid contents result in the increased hardness of a candy. The gummies in this study were prepared using bovine gelatin of strength 220 bloom, added to the formulation at 8.5% based on the final product, which is within the expected range for this type of product [48], while the water content ranged approximately from 25 to 30%, somewhat higher than expected. Despite our best effort to follow the same preparation protocol, final moisture content differed among formulations by up to 5% and we could not exclude its impact on the texture. The commercial product—“Ki-Ki gumioza” gummy bears (Kraš d.o.o, Zagreb, Croatia)—was analyzed as well to provide a reference in the sense that they are known to consumers. The declared ingredients for this product are glucose syrup, sugar, water, gelatin, citric acid and fruit juice (1%), while the nutrition facts are carbohydrate contents of 73%, of which sugars are 45%, protein content of 6.4% and salt content of 0.06%. By ingredients profile, the commercial sample closely resembled the HS_b_ formulation, as it had a lower gelatin content, assumed at the level of 6.4%, according to the protein content, whereas the water content was assumed at a level of 10–12% due to national market regulations for this type of product. The importance of analyzing a commercial sample is also shown by the difference in the analysis conditions of the previously reported data for similar products. Test parameters, as well as candy formulations, have a strong impact on the obtained results, making comparisons among different studies difficult. Texture parameters were measured using TPA analysis, the most commonly used method in the textural evaluation of sugar confections [5]. The analysis evaluated four basic independent parameters (hardness, cohesiveness, springiness and resilience) and one dependent parameter (chewiness) (Table 6). Adhesiveness was omitted from the results, since it could not be related to this type of product in a sensory way [49] and was biased by the oiling of the surface of the candies after removing them from the mold. The obtained results were analyzed for correlations (Table 7). It was shown that there were high positive correlations among the cohesiveness, resilience, springiness and chewiness parameters, whereas hardness did not significantly correlate to any of these parameters. The high positive correlations were maintained throughout the storage period. 

The hardness of the evaluated samples ranged from approximately 487 g (LS_b_) to 698 g (NS_inulin_) upon production and, generally, was somewhat lower than the commercial sample (approximately 721 g) (Table 6). With respect to their storage, only the LS_b_ and NS_b_ samples exhibited significant changes in hardness. The hardness for the LS_b_ sample was higher, whereas for NS_b_ it was lower. Additionally, NS_b_ showed significant changes over time for all of the evaluated texture parameters, which resulted from the observed crystallization on the surface of the candy. This was reflected in the increase in the values determining resilience, cohesiveness, springiness and gumminess, while the hardness value decreased. 

Resilience of the prepared candies (after production) ranged from approximately 55 g (HS_inulin_) to 92 g (HS_b_) (Table 6) and generally appeared to be a little higher than in the commercial sample (approximately 67 g). It was also observed that the inulin-containing samples exhibited the lowest resilience among samples. In gelled food systems, inulin has a synergistic effect with gelatin [50] and can possibly be used as a co-gelling agent. From our experience, during the cooking of the sugar syrup, the formulations containing inulin were much denser and more difficult to manipulate (e.g., incorporation of gelatin solution and molding) and exhibited a pasting process similar to that when cooking starch. However, the resulting gel seemed to exhibit attributes that were less elastic. Delgado and Bañón [34] investigated the impact of starch substitution with inulin in gelatin–starch jellies, which resulted in decreased gel strength and more deformable jellies, yet their elastic properties were more favorable than starch-containing reference. This implies that the impact of ingredients such as inulin can only be regarded in the context of the whole product. Also, it is important to note that inulins can differ in their characteristics which, in turn, can affect the texture of the product differently. In this study, over the storage period, the resilience significantly changed in only a few samples, including the mentioned NS_b_ sample but, in general, it can be noticed that the formulations were relatively stable with respect to resilience. Springiness has some similarities with resilience. In the prepared candies, it ranged between approximately 56 g (NS_b_ and HS_inulin_) and 68 g (HS_b_) (Table 6). Generally, the values were close to that of the commercial sample (68.2 g). As with resilience, inulin-containing samples exhibited somewhat lower values than the other formulations. Noticeably, the incorporation of inulin into formulations led to a relative loss of elasticity of the candies, as obtained instrumentally. 

Cohesiveness showed a high positive correlation with resilience and springiness, indicating that the elastic properties of the gummy candies were closely related or even determined by their highly cohesive structure. The cohesiveness ranged from 0.62 g (HS_inulin_) to 0.78 g (HS_b_, LS_b_) and was similar to the commercial sample (0.73) (Table 6). Again, inulin addition resulted in somewhat less cohesive formulations. With respect to storage, the cohesiveness of the candies was maintained, except for a few cases, such as HS_b_ NS_b_ and LS_FOS_, in which it significantly increased. Chewiness is calculated as a product of hardness, cohesiveness and springiness. Since chewiness was highly correlated with cohesiveness and springiness but not with hardness (Table 7), it can be concluded that the former parameters mostly influenced the chewiness of the produced candies. The prepared candies, in general, exhibited a lower chewiness than the commercial sample (605.45 g), except the HS_b_ formulation (650.44 g), ranging from approximately 372 to 559 g, with inulin-containing candies exhibiting among the lowest values (Table 6).

The behavior of the gelatin gels can be strongly influenced by the presence of co-solutes such as sugars, including polyols and/or other carbohydrates. The sugar profile and concentration are therefore important for the gelling properties of such systems. As explained by Wang and Hartel [48], the mechanical properties, which we perceive as texture, of candies prepared with hydrocolloids, depend on the interaction of the gelling agents, water and sugars, as well as the formation of junctions during the gelling process. Mechanisms supporting gelation in gelatin gels containing sugars include (i) the modification of hydrogen-bonding water structure, (ii) reduction in the amount of available water molecules due to the hydration of sugars and (iii) the exclusion of sugar molecules from the surface of polymeric hydrocolloid molecules, causing their aggregation [51]. In inulin candies, the polymeric nature of inulin and its reduced solubility at a high solids concentration might have disrupted the gelling behavior of the gelatin as the main gelling agent, making the resulting candy less elastic. For example, pectin–gelatin systems have shown variations in gelling behavior depending on their concentrations [52]. The addition of oligomeric prebiotics, on the other hand, did not show such a disruption, probably because they were more similar to the already present co-solutes, such as monosaccharidic and disccharidic sugars and glucose syrup. In addition, the presence of solubles from the extract might have influenced the gelling process as well, particularly concerning the content of cations. Gelling in multicomponent systems, such as formulations of herein studied candies is complex, including the interaction of different components, the water content of the gelled system, and the preparation method, including exposure to high temperatures and pH, so further investigations are needed to fully understand how each ingredient contributes to the change in texture. The ingredient quality should be accounted for as well, especially for gelatin and inulin. For now, from the obtained results, we can conclude that the elastic properties of the prepared candies were mostly determined by the highly correlated parameters of resilience, cohesiveness and springiness. Inulin exhibited a somewhat greater impact on the texture, across all sugar levels, than the other prebiotic ingredients. The texture of each formulation was mostly maintained during storage, while the strongest change was observed in NSb, which experienced crystallization. 

### 3.7. Sensory Evaluation

The prepared gummy bears were sensory evaluated in terms of visual appearance—transparency, taste—sweetness and bitterness and texture—hardness. Although children are usually the main consumers of gummy candies, in the present study, because of the incorporation of a bitter herbal extract, the prepared candies were not suited to younger populations. Therefore, the selected sensory panel was represented by adults aged 23–64 years old, previously experienced in sensory analysis. The gender uniformity of the panel (females) was purely coincidental and did not compromise the relevance of the obtained data since the panelists were experienced. The sensory evaluation was reduced to only five descriptors due to the large number of similar samples in the evaluation. The intensity of the perception of each of these attributes was evaluated on a 1–9 scale. In addition, each formulation was also evaluated for general acceptance, again on a 1–9 scale, taking into account the appearance, taste and texture. The obtained scores for all evaluated sensory parameters are presented in Table 8. Transparency is a characteristic property of gelatin gels, hence, also gummy candies [5]. However, the molding method, curing and finishing (e.g., shining) of the product can alter this property, as well as the presence of some ingredients in the formulation. In the present study, upon production, the candies were lightly oiled to prevent them from sticking together during storage and to simplify sampling during analyses. The samples, in general, exhibited relatively high transparency, except for the inulin-containing candies, which had an opaque appearance (1.6–2.5/9). It was also observed that the NS_b_ formulation was less transparent (3.7/9) than the NS formulations containing the prebiotics FOS (7.2/9) and XOS (6.9/9). Since the NS_b_ sample experienced significant changes during storage due to crystallization, the incorporation of FOS and XOS and a further reduction in the maltitol content might have improved the overall solubility of the ingredients and delayed crystallization. However, after storage, significant alterations in most of the NS formulations were visible (Figure 1), reflected in the loss of transparency. The exception was the NS_FOS_ sample in which the transparency increased, making its visual appearance more favorable. The transparency scores were higher after storage for other FOS- and XOS-containing formulations as well, indicating their, at least, neutral role in the visual aspect of the candy. The evaluated sweetness varied between 4.80 and 6.20/9, which is in the range of moderate to high intensity. It was observed that the NS formulations exhibited a higher sweetness intensity in comparison to HS and LS of the same added prebiotic group, including “basic” formulations as well. In the LS and NS formulations with prebiotics, a small amount of stevia powder was added to make up for the loss of sweetness due to the addition of prebiotics and polyols. This resulted in a somewhat more pronounced sweetness in these formulations. After 2 months of storage, in the formulations without the prebiotics, the sweetness intensity increased, while in the other formulations, except NS_inulin_, it generally decreased slightly, although not significantly. The highest evaluated sweetness after storage was evaluated in NS_inulin_. The HS_b_, HS_inulin_ and LS_inulin_ samples could not be evaluated for sensory attributes because of microbial contamination, observed as mold growth on the inside corners of the packaging. The contamination was somewhat expected because of the relatively high moisture content of the candies, which was a direct result of the preparation method and its limitations, as explained in Section 3.2. *Teucrium montanum* extracts (as well as extracts from other *Teucrium* species) are generally characterized by intense bitterness arising from the presence of bitter aromatic substances [53]. For this reason, the herb is used, in addition to treating respiratory conditions, for treating stomach problems [54]. The intense bitterness might be a limiting factor in the broader use of mountain germander and its bioactive potential. In the context of gummy candies, the sweetness of the formulations might hinder the bitterness of the extract and, hence, facilitate the acceptance of the extract. The evaluated bitterness intensity ranged from 4.10 to 5.10/9, which is in the moderate range, and the obtained scores were relatively uniformly distributed. It was interesting to observe that the bitterness intensity was reduced after storage in all samples, although shown as significant only in two samples. This indicates, perhaps, some sort of degradation of the bitter-carrying compounds over time or the change in their structure which disabled the development of the bitter taste. From the conducted analysis, it was not possible to reach a clear conclusion as to why the reduction in bitterness occurred. In *Teucrium* species, mainly diterpenoids have been identified as bitter-tasting compounds [54]. Specifically, in *T. montanum*, those include 19-acetylgnaphalin, montanin D, montanin B, teubotrin, montanin E and montanin H [55]. From a sensory aspect, the reduction in bitterness is certainly desirable in the context of gummy candy products. However, the experience of bitter taste and flavors in herbal-based products is somewhat expected, and since it was evaluated at a moderate level in the present study, it was acceptable. It is also noteworthy to mention that the panelists reported recognizing a honey-like flavor (resembling linden honey) in the prepared candies, although honey was not used as an ingredient. 

Sensory hardness was evaluated in the range of 4.40 to 6.00/9, dominantly in the moderate region. According to the TPA analysis, the prepared candies exhibited characteristic elastic and chewy properties very similar to those of a commercial product. Although some differences among the evaluated samples were found significant at a 0.05 level, sensory-wise, they might have been difficult to notice and distinguish. Depending on the product category and its predominant textural characteristic, the tolerance for the change in texture parameters can be very different [49]. For that reason, the evaluated sensory hardness was also correlated with TPA-determined parameters (Table 7). It was observed that sensory hardness positively correlated with instrumentally measured hardness (r = 0.467) after the production. It also exhibited a somewhat stronger correlation with other instrumentally evaluated parameters. Regarding the significant positive correlation of resilience and sensory hardness (r = 0.624), this implies that panelists might have experienced hardness in terms more appropriate for describing resilience, hence the persistence of the sample in regaining its initial height/shape. It also provides input into the mutual interconnection of these parameters, which makes them difficult to distinguish in a sensory evaluation. As with instrumental hardness, the sensory hardness correlated negatively with cohesiveness, springiness and chewiness. Samples that exhibited higher cohesiveness and springiness were sensory evaluated as less hard. The sensory hardness showed a negative and nonsignificant correlation with the instrumental hardness. Taking into account that the TPA parameters significantly changed in only a few cases, this might be the result of the lack of a true reference for hardness (i.e., a freshly prepared candy). It was impossible to remember the experienced hardness from the beginning of the study, and after 2 months any changes in the sensory hardness might have resulted in different arranging/alignment of the samples according to their hardness, since the panelists could only use intra-sample-based referencing. Therefore, a notable change in the correlation of measured and experienced hardness occurred. In addition, in three formulations (HS_b_, HS_inulin_ and LS_inulin_), microbial contamination (mold growth on the inside of the packaging) started to occur; hence, these samples no longer qualified for sensory evaluation, so the data range for obtaining correlations was reduced. After 2 months of storage, the experienced hardness was maintained, except for the HS_XOS_ sample, which was evaluated as more firm.

The general acceptance of the prepared candies was evaluated taking into account the combined visual, taste and textural properties of the candies. After the production, the highest scores were obtained for the “basic” formulations HS_b_ and LS_b_. This was somewhat expected, since these products were the most similar to the commercial ones. Candies with added prebiotics exhibited lower general acceptance scores, approximately 6/9. Among them, LS_FOS_ and NS_FOS_ were evaluated as the highest (6.70/9) and lowest (5.60/9) accepted, respectively. Interestingly, the opaque appearance of inulin-added candies did not negatively influence their general acceptance after production. However, the loss of their characteristic shape during storage, such as in the NS_inulin_ sample (Figure 1), or the occurrence of crystallization (NS_b_) significantly reduced the general acceptance score (approximately by half) (Table 8). After storage, the general acceptance of the candies increased (except the previously mentioned samples), although significantly only in the HS_FOS_ (7.10/9) and NS_XOS_ (6.30/9) samples. This could be due to the reduction in bitterness, along with the maintained textural parameters. The reduction in transparency in the NS formulations did not markedly affect their general acceptance; however, the very high transparency and lightness in color of FOS-containing formulations (HS and LS) might have favored their higher acceptance. 

## 4. Conclusions

In this study, the chemical (moisture, a_w_, pH, PhEG, and sugars), textural (TPA) and sensory (transparency, sweetness, bitterness, hardness, and general acceptance) stabilities of mountain germander extract-based gelatin gummy candies with varying sugar contents and fortified with prebiotics were evaluated. The proposed storage duration for the prepared candies is up to 1.5 (45 days) months, with respect to their relatively high moisture content and a_w_ values. Prolonged microbial stability could be achieved by reducing the moisture content; however, this would also affect the textural properties. The evaluated PhEG, deriving from the mountain germander extract, showed high stability during the candies’ preparation and storage. Echinacoside was found to be the dominant PhEG in the used plant material. With respect to appearance, the HS and LS formulations, regardless of the added prebiotics, kept their characteristic shape and appearance, while the NS formulations seemed less stable over time, since crystallization and surface malformation occurred in the NS_b_ and NS_inulin_ samples, respectively. The addition of FOS and XOS in the NS formulations, on the other hand, seemed to improve the stability of the formulations in terms of appearance. The color of the candies changed noticeably over time (ΔE approximately 4–5), as they appeared darker, especially XOS-containing samples, yet the color was still appropriate and desirable for a herbal-based type of product. The inulin-containing candies were completely opaque, whereas the NS formulations were less transparent in comparison to the LS and HS formulations. The sugar content analysis revealed the presence of sucrose and fructose in inulin and FOS, respectively, which should be accounted for, especially when considering sugar content claims. Sucrose inversion and hydrolysis of the prebiotics during production and storage occurred at low levels, if at all. The texture of the produced candies was characteristically elastic, having highly positively correlated TPA parameters of resilience, cohesiveness, springiness and chewiness. Although all formulations resembled the commercial product (although lower in hardness and chewiness), inulin addition had the most effect on the texture, which can be perceived as a relative loss in the elastic properties. In the sensory evaluation, most of the formulations were characterized by high transparency and moderate sweetness, bitterness and hardness. Changes in appearance in terms of the shape and surface, but not transparency, as well as bitterness intensity, had the strongest impact on the acceptance of the product. As the bitterness decreased slightly during storage, the general acceptance increased.

Among the evaluated formulations, the FOS- and XOS-containing ones have the highest potential for success as a functional product. These formulations showed high stability for all sugar level categories and across all evaluated parameters, including high general acceptance. The consumption of one serving size would contribute to approximately 32–40 mg of PhEG and up to 4.5 g of fiber. A slight advantage should be given to the XOS-fortified candies due to their lower effective dosage (from 3 g) and sustainability-oriented origin. Gelatin gummy bears have shown great potential in delivering bitter herbal extract and prebiotics by providing a familiar and highly acceptable food context, thus creating an innovative functional product. 

For future prospects, especially if considering the potential commercial stages of such candies, mountain germander extract standardization, with respect to PhEG and diterpenoid content, would be needed, and the PhEG-targeted bioactivity should be defined, as well as any potential toxicity of the diterpenoids. Furthermore, prebiotic properties of formulated candies should also be evaluated in the future. Another aspect to consider are modifications to achieve better microbiological stability of the product, along with the determination of shelf-life. 

## Figures and Tables

**Table 1:** Ingredients (in grams) used in gummy candy formulations.

Ingredient (g)	Formulations											
	HS_b_	LS_b_	NS_b_	HS_inulin_	LS_inulin_	NS_inulin_	HS_FOS_	LS_FOS_	NS_FOS_	HS_XOS_	LS_XOS_	NS_XOS_
Gelatin	30	30	30	30	30	30	30	30	30	30	30	30
Extract (gelatin)	60	60	60	60	60	60	60	60	60	60	60	60
Extract (syrup)	60	60	60	60	60	60	60	60	60	60	60	60
Sucrose	150	75	-	100	25		100	25	-	100	25	-
Glucose syrup	75	75	-	75	75	-	75	75	-	75	75	-
Xylitol	-	75	75	-	75	75	-	75	75	-	75	75
Maltitol	-	-	150	-	-	100	-	-	100	-	-	100
Inulin	-	-	-	50	50	50	-	-	-	-	-	-
FOS	-	-	-	-	-	-	50	50	50	-	-	-
XOS	-	-	-	-	-	-	-	-	-	50	50	50
Citric acid	3	3	3	3	3	3	3	3	3	3	3	3
Stevia	-	-	-	-	0.3	0.3	-	0.3	0.3	-	0.3	0.3

HS—“high-sugar” formulation; LS—“low-sugar” formulation; NS—“no-sugar” formulation; b—“basic” formulation, FOS—fructooligosaccharides; XOS—xylooligosaccharides.

**Table 2:** Dry matter, water activity and pH values in gummy candy formulations after production and 2 months of storage. Values in the same row denoted with the same letter are statistically different (*p* ≤ 0.05).

Sample	% Dry Matter		a_w_		pH	
	0 Months	2 Months	0 Months	2 Months	0 Months	2 Months
HS_b_	71.35 ± 0.47	71.97 ± 0.03	0.756 ± 0.001 ^a^	0.770 ± 0.003 ^a^	3.71 ± 0.05	3.68 ± 0.00
LS_b_	69.74 ± 0.73	69.73 ± 0.16	0.763 ± 0.002	0.765 ± 0.000	3.85 ± 0.01	3.86 ± 0.00
NS_b_	75.72 ± 0.14	75.96 ± 1.97	0.717 ± 0.004	0.725 ± 0.000	3.80 ± 0.01	3.83 ± 0.00
HS_inulin_	71.41 ± 0.40	70.85 ± 0.02	0.792 ± 0.002	0.792 ± 0.004	3.76 ± 0.07	3.76 ± 0.09
LS_inulin_	69.85 ± 0.64	69.91 ± 0.03	0.780 ± 0.009	0.788 ± 0.001	3.84 ± 0.01	3.88 ± 0.02
NS_inulin_	75.29 ± 0.24 ^b^	75.71 ± 0.34 ^b^	0.791 ± 0.009 ^c^	0.752 ± 0.001 ^c^	3.54 ± 0.07	3.56 ± 0.05
HS_FOS_	72.89 ± 0.94	72.73 ± 0.20	0.793 ± 0.002 ^d^	0.770 ± 0.001 ^d^	3.76 ± 0.04	3.75 ± 0.05
LS_FOS_	72.07 ± 0.56	72.40 ± 0.13	0.767 ± 0.005	0.759 ± 0.001	3.70 ± 0.02 ^e^	3.76 ± 0.03 ^e^
NS_FOS_	74.54 ± 0.19	75.16 ± 0.11	0.751 ± 0.003 ^f^	0.739 ± 0.001 ^f^	3.78 ± 0.07	3.77 ± 0.04
HS_XOS_	70.17 ± 0.47	70.74 ± 0.20	0.766 ± 0.003 ^g^	0.776 ± 0.001 ^g^	3.83 ± 0.00	3.81 ± 0.00
LS_XOS_	70.40 ± 0.18	70.91 ± 0.85	0.732 ± 0.000 ^h^	0.739 ± 0.001 ^h^	3.91 ± 0.00	3.89 ± 0.00
NS_XOS_	74.65 ± 0.14 ^i^	75.62 ± 0.03 ^i^	0.687 ± 0.001 ^j^	0.703 ± 0.002 ^j^	3.78 ± 0.01	3.79 ± 0.01

HS—“high-sugar” formulation; LS—“low-sugar” formulation; NS—“no-sugar” formulation; b—“basic” formulation; FOS—fructooligosaccharides; XOS—xylooligosaccharides.

**Table 3:** Contents of echinacoside, verbascoside and the sum of other phenylethanoid glycosides in gummy candy formulations after production and 2 months of storage. Values in the same row denoted with the same letter are statistically different (*p* ≤ 0.05).

	Echinacoside μg/g DM		Verbascoside μg/g DM		Other Phenylethanoid Glycosides μg/g DM *	
	0 Months	2 Months	0 Months	2 Months	0 Months	2 Months
HS_b_	929.82 ± 85.79 ^a^	765.67 ± 55.73 ^a^	127.75 ± 10.28 ^b^	106.76 ± 7.32 ^b^	813.69 ± 70.93 ^c^	645.68 ± 42.21 ^c^
LS_b_	915.75 ± 29.87	807.59 ± 25.37	127.93 ± 5.45	110.98 ± 3.28	822.62 ± 39.84 ^d^	677.27 ± 18.29 ^d^
NS_b_	899.19 ± 55.16	907.20 ± 14.44	123.49 ± 6.51	123.68 ± 1.47	787.76 ± 44.82	755.87 ± 9.84
HS_inulin_	877.97 ± 16.11	822.56 ± 31.06	120.54 ± 2.40	113.85 ± 4.24	691.16 ± 116.65	683.17 ± 24.94
LS_inulin_	824.93 ± 42.64	823.56 ± 37.50	121.00 ± 8.71	114.54 ± 5.05	782.23 ± 52.27	701.48 ± 28.09
NS_inulin_	705.86 ± 19.80 ^e^	654.00 ± 0.41 ^e^	96.48 ± 3.29 ^f^	87.13 ± 0.42 ^f^	617.46 ± 14.10 ^g^	539.07 ± 5.85 ^g^
HS_FOS_	781.03 ± 25.09	800.94 ± 53.66	109.40 ± 2.20	112.76 ± 7.66	689.46 ± 18.46	674.96 ± 42.20
LS_FOS_	743.12 ± 18.37	733.91 ± 52.17	103.18 ± 3.62	101.33 ± 7.90	662.77 ± 29.51	619.74 ± 44.11
NS_FOS_	676.89 ± 97.21	666.85 ± 24.22	101.64 ± 12.80	90.86 ± 4.03	592.56 ± 87.41	563.48 ± 23.70
HS_XOS_	815.42 ± 72.38	876.30 ± 21.19	113.38 ± 9.39	124.33 ± 3.51	735.09 ± 52.67	765.84 ± 17.03
LS_XOS_	854.74 ± 28.02	852.91 ± 29.81	120.89 ± 4.44	120.50 ± 4.61	773.12 ± 27.89	749.81 ± 26.02
NS_XOS_	744.20 ± 14.25	747.81 ± 7.06	103.52 ± 2.74	103.49 ± 1.18	668.47 ± 13.79	648.60 ± 5.78

* Expressed as echinacoside equivalents. HS—“high-sugar” formulation; LS—“low-sugar” formulation; NS—“no-sugar” formulation; b—“basic” formulation; FOS—fructooligosaccharides; XOS—xylooligosaccharides.

**Table 4:** Color parameters L* (lightness), a* (redness) and b* (yellowness) of the candy formulations after production and after 2 months of storage. ΔE was calculated for each formulation, with respect to a 0-month (after production) time point. Values within a row superscripted with the same letter are statistically different (*p* ≤ 0.05).

	0 Months			2 Months			
Sample	L*	a*	b*	L*	a*	b*	ΔE
HS_b_	29.80 ± 0.22	3.38 ± 0.39	13.66 ± 0.36 ^a^	29.59 ± 0.34	3.34 ± 0.47	8.20 ± 0.47 ^a^	5.50 ± 0.45
LS_b_	28.01 ± 0.62	4.33 ± 0.24	16.37 ± 0.97	26.18 ± 0.43	3.77 ± 0.34	12.89 ± 1.28	4.09 ± 1.00
NS_b_	32.40 ± 0.15 ^b^	3.46 ± 0.02	9.00 ± 0.04	43.38 ± 2.83 ^b^	2.55 ± 0.39	6.78 ± 1.00	11.28 ± 2.86
HS_inulin_	37.34 ± 0.09 ^c^	1.97 ± 0.19	12.47 ± 0.69	34.08 ± 0.56 ^c^	2.71 ± 0.11	11.68 ± 0.41	3.45 ± 0.65
LS_inulin_	26.69 ± 0.72	3.67 ± 0.15	12.14 ± 0.68	26.54 ± 1.67	4.03 ± 0.28	11.73 ± 1.65	2.26 ± 0.89
NS_inulin_	34.88 ± 0.21 ^d^	1.72 ± 0.06 ^e^	9.72 ± 0.22	33.49 ± 0.41 ^d^	2.60 ± 0.19 ^e^	9.36 ± 0.66	1.83 ± 0.32
HS_FOS_	29.36 ± 0.43 ^e^	4.35 ± 0.14	13.86 ± 0.34	26.32 ± 0.70 ^e^	4.51 ± 0.39	11.02 ± 0.96	4.35 ± 0.25
LS_FOS_	27.29 ± 0.42	4.23 ± 0.21	17.69 ± 1.10 ^f^	26.82 ± 1.28	4.11 ± 0.38	12.43 ± 1.43 ^f^	5.45 ± 1.44
NS_FOS_	31.09 ± 0.39	3.70 ± 0.19	13.54 ± 0.95	30.38 ± 1.37	2.89 ± 0.47	11.32 ± 1.18	3.00 ± 0.77
HS_XOS_	29.32 ± 0.39 ^g^	5.31 ± 0.19	12.95 ± 0.41 ^h^	26.58 ± 0.73 ^g^	5.79 ± 0.48	7.84 ± 0.79 ^h^	5.94 ± 0.27
LS_XOS_	28.61 ± 1.22 ^i^	4.60 ± 0.65 ^j^	12.74 ± 1.83	23.23 ± 1.21 ^i^	6.90 ± 0.46 ^j^	12.60 ± 1.10	5.96 ± 1.26
NS_XOS_	29.36 ± 0.16 ^k^	4.59 ± 0.16	12.55 ± 0.35 ^l^	28.26 ± 0.91 ^k^	4.93 ± 0.16	9.05 ± 0.27 ^l^	3.80 ± 0.23

HS—“high-sugar” formulation; LS—“low-sugar” formulation; NS—“no-sugar” formulation; b—“basic” formulation; FOS—fructooligosaccharides; XOS—xylooligosaccharides.

**Table 5:** Contents of different sugars in the ingredients and prepared gummy candies after production and 2 months of storage (dmb—dry matter basis; n.d.—not detected; suc—sucrose; malt—maltose; glc—glucose; fru—fructose). Values within a column superscripted with the same letter are statistically different (*p* ≤ 0.05).

Ingredient	Sucrose (%)	Maltose (%)	Glucose (%)	Fructose (%)	Xylose (%)
Glucose syrup	n.d.	11.04	15.36	n.d.	n.d.
Inulin	6.59	n.d.	n.d.	n.d.	n.d.
FOS		n.d.	n.d.	4.20	n.d.
XOS	n.d.	n.d.	n.d.	n.d.	n.d.
Sample/sugar	Suc+Malt (% dmb)	Glc (% dmb)	Fru (% dmb)	Xylitol (% dmb)	Maltitol (% dmb)
	0 months				
HS_b_	65.85 ± 1.12	6.69 ± 0.71	1.14 ± 0.07 ^j^	n.d.	n.d.
LS_b_	33.93 ± 0.47 ^a^	6.16 ± 0.22	n.d. ^k^	32.93 ± 0.00	n.d.
NS_b_	n.d.	n.d.	n.d.	37.50 ± 0.13 ^p^	48.53 ± 0.22 ^r^
HS_inulin_	43.38 ± 1.02	6.54 ± 0.39 ^f^	1.58 ± 0.16	n.d.	n.d.
LS_inulin_	15.19 ± 0.06 ^b^	5.61 ± 0.07 ^g^	n.d.^l^	33.34 ± 0.09	n.d.
Ns_inulin_	1.30 ± 0.25 ^c^	n.d.	n.d.	31.69 ± 5.78	34.07 ± 1.53 ^s^
HS_FOS_	42.78 ± 0.83	10.40 ± 0.10^h^	3.17 ± 0.17 ^m^	n.d.	n.d.
LS_FOS_	14.27 ± 0.54 ^d^	9.68 ± 0.03	2.23 ± 0.25	33.53 ± 0.70	n.d.
NS_FOS_	0.79 ± 0.04	n.d.	n.d. ^n^	30.66 ± 2.43	38.17 ± 1.69
HS_XOS_	50.33 ± 0.23	5.70 ± 0.35	n.d. ^o^	n.d.	n.d.
LS_XOS_	16.24 ± 0.19 ^e^	5.65 ± 0.09 ^i^	n.d.	32.86 ± 0.15	n.d.
NS_XOS_	n.d.	n.d.	n.d.	28.48 ± 2.04	38.12 ± 1.12
	2 months				
HS_b_	59.53 ± 2.86	6.34 ± 0.98	1.36 ± 0.02 ^j^	n.d.	n.d.
LS_b_	31.65 ± 0.39 ^a^	5.40 ± 0.41	0.24 ± 0.01 ^k^	32.27 ± 0.45	n.d.
NS_b_	n.d.	n.d.	n.d.	36.46 ± 0.12 ^p^	45.69 ± 0.33 ^r^
HS_inulin_	42.53 ± 0.55	5.48 ± 0.20 ^f^	1.15 ± 0.03	n.d.	n.d.
LS_inulin_	13.91 ± 0.05 ^b^	4.87 ± 0.03 ^g^	0.24 ± 0.00 ^l^	33.34 ± 1.21	n.d.
Ns_inulin_	≤0.5^c^	n.d.	n.d.	29.93 ± 0.16	37.75 ± 0.24 ^s^
HS_FOS_	40.02 ± 0.34	8.59 ± 0.34 ^h^	2.41 ± 0.04 ^m^	n.d.	n.d.
LS_FOS_	12.74 ± 0.35 ^d^	8.90 ± 0.82	2.00 ± 0.10	32.68 ± 0.34	n.d.
NS_FOS_	0.33 ± 0.00	n.d.	0.82 ± 0.03 ^n^	30.21 ± 0.48	38.54 ± 0.82
HS_XOS_	49.46 ± 1.27	5.95 ± 0.12	0.82 ± 0.15 ^o^	n.d.	n.d.
LS_XOS_	13.59 ± 0.21^e^	4.33 ± 0.06 ^i^	n.d.	32.59 ± 0.51	n.d.
NS_XOS_	n.d.	n.d.	n.d.	30.43 ± 0.60	37.17 ± 0.16

n.d.—not detected; HS—“high-sugar” formulation; LS—“low-sugar” formulation; NS—“no-sugar” formulation; b—“basic” formulation; FOS—fructooligosaccharides; XOS—xylooligosaccharides.

**Table 6:** Parameters of the TPA analysis of the gummy bears after production and 2 months of storage. Samples that were statistically different (*p* ≤ 0.05) for each evaluated TPA parameter (columns) are denoted with the same letter.

	Sample	Hardness (g)	Resilience (g)	Cohesiveness (g)	Springiness (g)	Chewiness (g)
0 months	HS_b_	558.81 ± 1.86	92.13 ± 3.09	0.78 ± 0.01 ^f^	68.18 ± 2.27	650.44 ± 73.12
	LS_b_	486.81 ± 30.77 ^a^	87.25 ± 2.16	0.78 ± 0.00	63.64 ± 0.00	525.91 ± 10.17
	NS_b_	591.76 ± 26.38 ^b^	68.54 ± 7.03 ^c^	0.67 ± 0.03 ^g^	56.06 ± 3.47 ^i^	372.28 ± 46.36 ^l^
	HS_inulin_	569.80 ± 43.70	54.98 ± 0.12	0.62 ± 0.01	56.06 ± 1.31	386.86 ± 24.17
	LS_inulin_	564.94 ± 17.18	79.45 ± 1.06 ^d^	0.70 ± 0.00	58.33 ± 1.31	435.67 ± 6.25
	NS_inulin_	697.52 ± 107.85	77.70 ± 1.28	0.71 ± 0.02	60.61 ± 1.31	481.49 ± 37.27 ^m^
	HS_FOS_	640.03 ± 46.74	85.25 ± 1.93	0.76 ± 0.02	64.39 ± 1.31	558.72 ± 48.24
	LS_FOS_	593.23 ± 44.93	84.10 ± 2.24	0.74 ± 0.00 ^h^	61.36 ± 0.00 ^j^	491.43 ± 22.75 ^n^
	NS_FOS_	624.36 ± 100.72	90.20 ± 0.27	0.74 ± 0.00	63.64 ± 2.27	527.47 ± 33.92
	HS_XOS_	495.69 ± 33.19	87.75 ± 1.88	0.75 ± 0.01	65.15 ± 1.31	512.03 ± 9.21
	LS_XOS_	554.52 ± 10.67	86.29 ± 1.95	0.74 ± 0.01	64.39 ± 1.31 ^k^	504.49 ± 11.02
	NS_XOS_	593.34 ± 25.65	89.51 ± 0.09 ^e^	0.72 ± 0.01	63.64 ± 0.00	502.03 ± 11.61
	Commercial product	721.49 ± 13.74	66.73 ± 1.50	0.73 ± 0.00	68.18 ± 2.27	605.45 ± 28.41
2 months	HS_b_	721.82 ± 91.14	83.67 ± 2.53	0.82 ± 0.02 ^f^	65.15 ± 1.31	616.15 ± 91.69
	LS_b_	566.43 ± 20.58 ^a^	84.31 ± 1.35	0.78 ± 0.01	64.39 ± 1.31	497.34 ± 33.25
	NS_b_	486.07 ± 20.48 ^b^	90.15 ± 1.10 ^c^	0.76 ± 0.01 ^g^	65.91 ± 0.00 ^i^	545.47 ± 17.34 ^l^
	HS_inulin_	618.22 ± 20.29	59.28 ± 3.64	0.63 ± 0.04	56.06 ± 2.62	376.70 ± 54.99
	LS_inulin_	570.95 ± 26.75	83.02 ± 1.43 ^d^	0.72 ± 0.02	61.36 ± 2.27	472.46 ± 34.73
	NS_inulin_	686.95 ± 73.78	73.77 ± 3.18	0.68 ± 0.07	57.58 ± 7.31	387.47 ± 3.89 ^m^
	HS_FOS_	642.56 ± 51.92	90.28 ± 5.34	0.76 ± 0.02	65.15 ± 2.62	569.23 ± 64.34
	LS_FOS_	579.51 ± 43.80	88.81 ± 0.54	0.76 ± 0.01 ^h^	65.15 ± 1.31 ^j^	542.02 ± 15.42 ^n^
	NS_FOS_	620.88 ± 51.13	85.85 ± 1.94	0.74 ± 0.01	62.12 ± 1.31	518.85 ± 52.78
	HS_XOS_	552.45 ± 12.20	83.30 ± 3.85	0.75 ± 0.00	62.88 ± 1.31	467.80 ± 10.71
	LS_XOS_	566.00 ± 25.12	85.61 ± 1.21	0.73 ± 0.01	62.12 ± 1.31 ^k^	478.18 ± 22.52
	NS_XOS_	592.82 ± 14.95	83.35 ± 1.23 ^e^	0.72 ± 0.01	62.12 ± 1.31	491.01 ± 29.31

HS—“high-sugar” formulation; LS—“low-sugar” formulation; NS—“no-sugar” formulation; b—“basic” formulation; FOS—fructooligosaccharides; XOS—xylooligosaccharides.

**Table 7:** Pearson correlations for texture parameters obtained by TPA and sensory hardness, after production and 2 months of storage. Significant correlations at the levels of 0.05 and 0.01 (2-tailed) are denoted with asterisks (* and **, respectively).

		Hardness	Resilience	Cohesiveness	Springiness	Chewiness	Sensory Hardness
0 months	Hardness	1	−0.141	−0.232	−0.209	−0.075	0.467
	Resilience		1	0.919 **	0.882 **	0.819 **	0.624 *
	Cohesiveness			1	0.893 **	0.877 **	−0.688 *
	Springiness				1	0.950 **	−0.505
	Chewiness					1	−0.521
	Sensory hardness						1
2 months	Hardness	1	−0.315	−0.002	−0.278	0.055	−0.125
	Resilience		1	0.798 **	0.899 **	0.783 **	0.043
	Cohesiveness			1	0.918 **	0.900 **	−0.010
	Springiness				1	0.909 **	0.023
	Chewiness					1	−0.031
	Sensory hardness						1

**Table 8:** Sensory evaluation scores for the gummy candy formulations after production and 2 months of storage. Samples in the same column that were statistically different (*p* ≤ 0.05) for each evaluated parameter are denoted with the same letter.

Sample/Parameter	Transparency	Sweetness	Bitterness	Hardness	General Acceptance
	0 months				
HS_b	8.50 ± 0.67 ^a^	5.80 ± 0.75	4.30 ± 1.42	4.90 ± 0.83	7.50 ± 0.92
LS_b	8.30 ± 0.46	5.20 ± 0.75	5.10 ± 1.45	4.40 ± 0.80	7.20 ± 0.75
NS_b	3.70 ± 0.64 ^b^	6.20 ± 0.98	4.30 ± 1.62	6.00 ± 0.77	6.10 ± 1.04 ^m^
HS_inulin	1.60 ± 0.49	5.00 ± 1.00	4.10 ± 1.37	6.20 ± 1.54	6.00 ± 1.00
LS_inulin	2.30 ± 0.46	4.80 ± 0.87	4.50 ± 1.12	5.00 ± 0.63	6.10 ± 0.54
NS_inulin	2.50 ± 0.50 ^c^	5.60 ± 1.02 ^i^	4.30 ± 1.10 ^j^	6.00 ± 1.90	6.00 ± 0.89 ^n^
HS_FOS	6.70 ± 1.00	5.30 ± 1.00	4.30 ± 1.49	5.70 ± 0.64	6.10 ± 0.54 ^o^
LS_FOS	6.80 ± 0.98 ^d^	5.70 ± 0.78	4.30 ± 1.19	5.20 ± 0.98	6.70 ± 0.90
NS_FOS	7.20 ± 0.40 ^e^	6.20 ± 0.60	3.90 ± 1.30	5.00 ± 0.45	5.60 ± 1.20
HS_XOS	5.80 ± 1.08 ^f^	5.10 ± 0.94	4.90 ± 1.58 ^k^	5.70 ± 0.78 ^l^	6.40 ± 1.02
LS_XOS	8.10 ± 0.54 ^g^	5.20 ± 1.08	4.40 ± 1.11	4.90 ± 0.94	6.00 ± 0.89 ^p^
NS_XOS	6.90 ± 0.70 ^h^	6.20 ± 0.75	4.40 ± 1.43	5.80 ± 0.75	6.00 ± 0.63
	2 months				
HS_b	7.10 ± 0.54 ^a^	n.d.	n.d.	n.d.	n.d.
LS_b	8.60 ± 0.49	5.80 ± 0.75	4.00 ± 1.34	4.90 ± 1.04	7.60 ± 0.66
NS_b	1.10 ± 0.30 ^b^	6.00 ± 0.89	3.30 ± 1.00	5.90 ± 0.83	3.10 ± 1.81 ^m^
HS_inulin	1.80 ± 0.75	n.d.	n.d.	n.d.	n.d.
LS_inulin	2.30 ± 0.78	n.d.	n.d.	n.d.	n.d.
NS_inulin	1.10 ± 0.30 ^c^	6.56 ± 0.83 ^i^	2.30 ± 1.10 ^j^	5.40 ± 1.67	3.00 ± 1.18 ^n^
HS_FOS	7.60 ± 0.49	4.90 ± 0.54	4.00 ± 1.00	6.50 ± 1.20	7.10 ± 0.70 ^o^
LS_FOS	8.20 ± 0.75 ^d^	5.50 ± 0.67	3.30 ± 1.00	4.40 ± 1.50	7.10 ± 0.83
NS_FOS	3.80 ± 1.08 ^e^	5.60 ± 0.66	3.30 ± 1.25	5.20 ± 1.33	6.00 ± 0.77
HS_XOS	7.50 ± 0.81 ^f^	5.20 ± 0.60	3.30 ± 0.78 ^k^	7.20 ± 1.08 ^l^	6.70 ± 0.64
LS_XOS	8.70 ± 0.46 ^g^	5.10 ± 1.41	3.60 ± 0.66	5.70 ± 1.00	6.30 ± 0.64 ^p^
NS_XOS	5.30 ± 1.27 ^h^	5.70 ± 0.46	3.50 ± 0.92	5.60 ± 1.20	6.60 ± 0.66

n.d.—not determined due to spoilage. HS—“high-sugar” formulation; LS—“low-sugar” formulation; NS—“no-sugar” formulation; b—“basic” formulation; FOS—fructooligosaccharides; XOS—xylooligosaccharides.

## Data Availability

Data are contained within the article.

## References

[1] Dille M.J., Hattrem M.N., Draget K.I. (2018). Bioactively Filled Gelatin Gels; Challenges and Opportunities. Food Hydrocoll..

[2] Pickford E.F., Jardine N.J., Gibson G.R., Williams C.M. (2000). Functional Confectionery. Functional Foods—Concepts to Products.

[3] Food and Beverage Insider Chewy Candy Sales Continue to Climb (Bradford, K.). https://www.foodbeverageinsider.com/confectionery/chewy-candy-sales-continue-to-climb.

[4] Mandura A., Šeremet D., Ščetar M., Vojvodić Cebin A., Belščak-Cvitanović A., Komes D. (2020). Physicochemical, Bioactive, and Sensory Assessment of White Tea-Based Candies During 4-Months Storage. J. Food Process. Preserv..

[5] Gunes R., Palabiyik I., Konar N., Toker O.S. (2022). Soft Confectionery Products: Quality Parameters, Interactions with Processing and Ingredients. Food Chem..

[6] Baziwane D., He Q. (2003). Gelatin: The Paramount Food Additive. Food Rev. Int..

[7] Dille M.J., Haug I.J., Draget K.I., Phillips G.O., Williams P.A. (2021). Gelatin and Collagen. Handbook of Hydrocolloids.

[8] Konar N., Gunes R., Palabiyik I., Toker O.S. (2022). Health Conscious Consumers and Sugar Confectionery: Present Aspects and Projections. Trends Food Sci. Technol..

[9] Liu D., Nikoo M., Boran G., Zhou P., Regenstein J.M. (2015). Collagen and Gellatine. Annu. Rev. Food Sci. Technol..

[10] Rawson E.S., Miles M.P., Larson-Meyer D.E. (2018). Dietary Supplements for Health, Adaptation, and Recovery in Athletes. Int. J. Sport Nutr. Exerc..

[11] Periche A., Heredia A., Escriche A., Andrés A., Castelló M.L. (2014). Optical, Mechanical and Sensory Properties of Based-Isomaltulose Gummy Confections. Food Biosci..

[12] Mandura Jarić A., Čikoš A., Pocrnić M., Aladić K., Jokić S., Šeremet D., Vojvodić Cebin A., Komes D. (2023). *Teucrium montanum* L.—Unrecognized Source of Phenylethanoid Glycosides: Green Extraction Approach and Elucidation of Phenolic Compounds via NMR and UHPLC-HR MS/MS. Antioxidants.

[13] Nastić N., Švarc-Gajić J., Delerue-Matos C., Morais S., Barroso M.F., Moreira M.M. (2018). Subcritical Water Extraction of Antioxidants from Mountain Germander (*Teucrium montanum* L.). J. Supercrit. Fluids.

[14] Stanković M.S., Niciforović M., Topuzović M., Solujić S. (2011). Total Phenolic Content, Flavonoid Concentrations and Antioxidant Activity, of the Whole Plant and Plant Parts Extracts from *Teucrium montanum* L. var. *Montanum*, F. *Supinum* (L.) Reichenb. Biotechnol. Biotechnol. Equip..

[15] Šeremet D., Vugrinec K., Petrović P., Butorac A., Kuzmić S., Vojvodić Cebin A., Mandura A., Lovrić M., Pjanović R., Komes D. (2022). Formulation and Characterization of Liposomal Encapsulated Systems of Bioactive Ingredients from Traditional Plant Mountain Germander (*Teucrium montanum* L.) for the Incorporation into Coffee Drinks. Food Chem..

[16] Tian X.Y., Li M., Lin T., Qiu Y., Zhu Y.T., Li X.L., Tao W.D., Wang P., Ren X.X., Chen L.P. (2021). A Review on the Structure and Pharmacological Activity of Phenylethanoid Glycosides. Eur. J. Med. Chem..

[17] Bernardi M., Ghaani M.R., Bayazeid O. (2021). Phenylethanoid Glycosides as a Possible COVID-19 Protease Inhibitor: A Virtual Screening Approach. J. Mol. Model..

[18] Cheohen C.F.A.R., Esteves M.E.A., da Fonseca T.S., Leal C.M., Assis F.L.F., Campos M.F., Rebelo R.S., Allonso D., Leitão G.G., da Silva M.L. (2023). *In silico* Screening of Phenylethanoid Glycosides, a Class of Pharmacologically Active Compounds as Natural Inhibitors of SARS-CoV-2 Proteases. Comput. Struct. Biotechnol. J..

[19] Zumbé A., Lee A., Storey D. (2001). Polyols in Confectionery: The Route to Sugar-Free, Reduced Sugar and Reduced Calorie Confectionery. Br. J. Nutr..

[20] Livesey G. (2003). Health Potential of Polyols as Sugar Replacers, with Emphasis on Low Glycaemic Properties. Nutr. Res. Rev..

[21] Cai L., Feng J., Regenstein J., Lv Y., Li J. (2017). Confectionery gels: Effects of Low Calorie Sweeteners on the Rheological Properties and Microstructure of Fish Gelatin. Food Hydrocoll..

[22] Franck A., Gibson G.R., Roberfroid M.B. (2008). Food Applications of Prebiotics. Handbook of Prebiotics.

[23] Gok S., Toker O.S., Palabiyik I., Konar N. (2020). Usage Possibility of Mannitol and Soluble Wheat Fiber in Low Calorie Gummy Candies. LWT-Food Sci. Technol..

[24] Lele V., Ruzauskas M., Zavistanaviciute P., Laurusiene R., Rimene G., Kiudulaite D., Tomkeviciute J., Nemeikstyte J., Stankevicius R., Bartkiene E. (2018). Development and Characterization of the Gummy–Supplements, Enriched with Probiotics and Prebiotics. CyTA-J. Food.

[25] Amorim C., Silvero S.C., Prather K.L.J., Rodrigues L.R. (2019). From Lignocellulosic Residues to Market: Production and Commercial Potential of Xylooligosaccharides. Biotechnol. Adv..

[26] Palaniappan A., Antony U., Emmambux M.N. (2021). Current Status of Xylooligosaccharides: Production, Characterization, Health Benefits and Food Application. Trends Food Sci. Technol..

[27] Shandong Longlive Biotechnology Co., Ltd. http://www.longlivegroup.com/col.jsp?id=104.

[28] Vojvodić Cebin A., Ralet M.C., Vigouroux J., Karača S., Martinić A., Komes D., Bonnin E. (2021). Valorisation of Walnut Shell and Pea Pod as Novel Sources for the Production of Xylooligosaccharides. Carbohydr. Polym..

[29] Samanta A.K., Jayapal N., Jayaram C., Roy S., Kolte A.P., Senani S., Sridhar M. (2015). Xylooligosaccharides as Prebiotics from Agricultural By-Products: Production and Applications. Bioact. Carbohydr. Diet. Fibre.

[30] Cruz-Guerrero A., Gómez-Ruiz L., Guzmán-Rodríguez F., Jafari S.M., Rashidinejad A., Simal-Ganara J. (2023). Xylooligosaccharides. Handbook of Food Bioactive Ingredients: Properties and Appplications.

[31] Seifert S., Watzl B., Gibson G.R., Roberfroid M.B. (2008). Prebiotics and the Immune System: Review of Experimental and Human Data. Handbook of Prebiotics.

[32] Van der Abbeele P., Duysburgh C., Jiang T.A., Rebaza M., Pinheiro I., Marzorati M. (2018). A Combination of Xylooligosaccharides and a Polyphenol Blend Affect Microbial Composition and Activity in the Distal Colon Exerting Immunomodulating Properties on Human Cells. J. Funct. Foods.

[33] Padmore J.M., Helrich K. (1990). Animal Feed—AOAC official method 930.15—Moisture in Animal Feed. Official Methods of Analysis.

[34] Delgado P., Bañón S. (2017). Effects of Replacing Starch by Inulin on Physicochemical, Texture and Sensory Characteristics of Gummy Jellies. CyTA-J. Food.

[35] Ergun R., Lietha R., Hartel R.W. (2010). Moisture and Shelf Life in Sugar Confections. Crit. Rev. Food Sci. Nutr..

[36] Efe N., Dawson P. (2022). A Review: Sugar-based Confectionery and the Importance of Ingredients. Eur. J. Agric. Food Sci..

[37] Plotnikova V., Zharkova I.M., Magomedov G.O., Magomedov M.G., Khvostov A.A., Miroshnichenko E.N. (2021). Forecasting and Quality Control of Confectionery Products with the Use of “Water Activity” Indicator. IOP Conf. Ser. Earth Environ. Sci..

[38] Burey P., Bhandari B.R., Rutgers R.P.G., Halley P.J., Torley P.J. (2009). Confectionery gels: A Review on Formulation, Rheological and Structural Aspects. Int. J. Food Prop..

[39] Wang W., Jiang S., Zhao Y., Zhu G. (2023). Echinacoside: A Promising Active Natural Products and Pharmacological Agents. Pharmacol. Res..

[40] FDA-U.S. Food and Drug Administration FDA, Serving Size Updates on the New Nutrition Facts Label. https://www.fda.gov/food/new-nutrition-facts-label/serving-size-updates-new-nutrition-facts-label.

[41] Mokrzycki W.S., Tatol M. (2011). Colour Difference ΔE—A Survey. Mach. Graph. Vis..

[42] Stokes M., Fairchild M.D., Berns R.S. (1992). Precision Requirements for Digital Color Reproduction. ACM Trans. Graph..

[43] EFSA Nutrition Claims. https://food.ec.europa.eu/safety/labelling-and-nutrition/nutrition-and-health-claims/nutrition-claims_en.

[44] Finegold S.M., Li Z., Summanen P.H., Downes J., Thames G., Corbett K., Dowd S., Krakde M., Heberde D. (2014). Xylooligosaccharide Increases Bifidobacteria but not Lactobacilli in Human Gut Microbiota. Food Funct..

[45] EFSA, Panel on Dietetic Products, Nutrition, and Allergies (NDA) (2010). Scientific opinion on dietary reference values for carbohydrates and dietary fibre. EFSA J..

[46] Lee W., Spiekerman C., Heima M., Eggertsson H., Ferretti G., Milgrom P., Nelson S. (2015). The Effectiveness of Xylitol in a School-based Cluster-randomized Clinical Trial. Caries Res..

[47] Ly K.A., Riedy C.A., Milgrom P., Rothen M., Roberts M.C., Zhou L. (2008). Xylitol Gummy Bear Snacks: A School-based Randomized Clinical Trial. BMC Oral Health.

[48] Wang R., Hartel R.W. (2022). Confectionery gels: Gelling Behavior and Gel Properties of Gelatin in Concentrated Sugar Solutions. Food Hydrocoll..

[49] Szczesniak A.S. (2002). Texture is a Sensory Property. Food Qual. Prefer..

[50] Roberfroid M.B. (2005). Introducing Inulin-type Fructans. Br. J. Nutr..

[51] Avallone P.R., Romano M., Sarrica A., Delmonte M., Pasquino R., Grizzuti N. (2022). Effect of Sugars on Gelation Kinetics of Gelatin Gels. Fluids.

[52] DeMars L.L., Ziegler G.R. (2001). Texture and Structure of Gelatin/Pectin-based Gummy Confections. Food Hydrocoll..

[53] Ersoy E., Süvari G., Ercan S., Özkan E.E., Karahan S., Tuncay E.A., Cantürk Y.Y., Kara E.M., Zengin G., Boğa M. (2023). Towards a Better Understanding of Commonly Used Medicinal Plants from Turkiye: Detailed Phytochemical Screening and Biological Activity Studies of Two *Teucrium* L. Species with *in vitro* and *in silico* Approach. J. Ethnopharmacol..

[54] Jurišić Grubešić R., Kremer D., Vladimir-Knežević S., Vuković Rodríguez J. (2012). Analysis of Polyphenols, Phytosterols, and Bitter Principles in *Teucrium* L. Species. Cent. Eur. J. Biol..

[55] Malakov P.Y., Papanov G.Y., Boneva I.M. (1992). Neo-clerodane Diterpenoids from *Teucrium montanum*. Phytochemistry.

